# Interoception is associated with the impact of eye contact on spontaneous facial mimicry

**DOI:** 10.1038/s41598-020-76393-8

**Published:** 2020-11-16

**Authors:** Masahiro Imafuku, Hirokata Fukushima, Yuko Nakamura, Masako Myowa, Shinsuke Koike

**Affiliations:** 1 Faculty of Education, Musashino University, 1-1-20 Shin-machi, Nishitokyo-shi, Tokyo 202-8585 Japan; 2 Faculty of Sociology, Kansai University, 3-3-35 Yamate-cho, Suita, Osaka 564-8680 Japan; 3 Center for Evolutionary Cognitive Sciences, Graduate School of Arts and Sciences, The University of Tokyo, 3-8-1 Komaba, Meguro-ku, Tokyo 153-8902 Japan; 4 Center for Integrative Science of Human Behavior, Graduate School of Arts and Sciences, The University of Tokyo, 3-8-1 Komaba, Meguro-ku, Tokyo 153-8902 Japan; 5 Graduate School of Education, Kyoto University, Kyoto, Japan; 6 University of Tokyo Institute for Diversity & Adaptation of Human Mind (UTIDAHM), 3-8-1 Komaba, Meguro-ku, Tokyo 153-8902 Japan; 7 The International Research Center for Neurointelligence (WPI-IRCN) at The University of Tokyo Institutes for Advanced Study (UTIAS), 7-3-1 Hongo, Bunkyo-ku, Tokyo 113-8654 Japan

**Keywords:** Insula, Human behaviour

## Abstract

Interoception (perception of one’s own physiological state) has been suggested to underpin social cognition, although the mechanisms underlying this association remain unclear. The current study aimed to elucidate the relationship between interoception and two factors underlying social cognition: self-other boundary and sensitivity to social cues. We measured performance in a heartbeat perception task as an index of interoceptive accuracy (IAc), the frequency of spontaneous facial mimicry (SFM) as an index of self-other boundary, and the degree of the effect of eye contact on SFM (difference in SFM between conditions in which models’ eyes were directed to and averted from participants) as an index of social-cue sensitivity, and tested correlations among these measures. The results revealed that IAc and SFM were positively correlated only in the direct gaze condition. The extent of the effect of eye contact on SFM (difference in frequency between direct vs. averted conditions) was positively correlated with IAc. These overall findings were also observed in separate analyses of male and female participant groups, supporting the robustness of the findings. The results suggest that interoception is related to sensitivity to social cues, and may also be related to the self-other boundary with modulation by social context.

## Introduction

### Relationship between interoception and social cognition

Many researchers have proposed that interoception, the sense of the physiological state of the inner body, is crucially related to emotional awareness^1–3^ and self-consciousness^4,5^. In addition, whether and how interoception is related to *social* cognition (i.e., understanding others, such as empathy and mentalising) have also been topics of growing research interest^6–9^. Several behavioural and neuroscientific studies have suggested associations between interoception, empathy and mentalising^9–12^ (but see^6^). Most of these previous studies have explained the association between interoception and social cognitive abilities by assuming the following syllogism: sensitivity to the bodily state (interoception) and the mental/affective states of one’s own are correlated, and sensitivity to the mental states of the self and others are correlated; therefore, sensitivity to bodily states of the self and mental states of others are correlated. Although this logic captures the essence of the issue, it may be overly simple for further elucidating the mechanisms underlying the relationship between interoception and social cognition. Social processing is underpinned by not only perceiving the mental/affective states of others, but also other functions, such as motor recognition, adjustment of the self-other boundary, and detecting socially relevant stimuli^13,14^. Whether and how these functions are associated with interoception remain unclear. Against this background, the present study investigated the relationship between interoceptive sensitivity and two factors underlying social cognition: the degree of self-other boundary (interpersonal overlap/distinction), and sensitivity to socially relevant signals.

### Self-other boundary

In the current study, the term “self-other boundary” refers to the degree of overlap or distinction in processing of the self and others, which includes both conscious and unconscious processes of self and other. The current study focuses on the latter component by measuring embodied reactions to others. Social cognition is essentially a process of representing others within the self. Understanding others is thought to be primarily realised by some sort of matching and sharing process on the body image and/or mental states between self and other. Self-other overlapping is suggested to facilitate understanding others and empathic responses^15,16^. At the same time, without self-other distinction, one may confuse the experiences of the self and others, leading to situations involving an incorrect understanding of others, or being uncontrollably influenced by others’ negative emotions^13^. Cognitive studies have recently suggested that the ability to perform self-other distinction is positively associated with the degree of empathic response^13^, the ability of perspective taking^17^, and self-other control^18^. As such, the adjustability or balance between self-other overlap and distinction (i.e. self-other boundary) is important factor for individuals’ social skills and traits.

The interoceptive factors that potentially modulate the self-other boundary have been examined in several recent studies. Some of these studies used an “imitation inhibition” (or “automatic imitation”) reaction time task paradigm. In this paradigm, participants’ reactions can be distracted by visual stimuli showing another person’s actions, and the amount of distraction is measured as an index of the self-other boundary^19,20^. Ainley et al. reported a positive correlation between performance on the heartbeat counting task and distractibility in the automatic imitation task, suggesting that individuals with greater interoceptive sensitivity have greater susceptibility to others^21^. Sowden et al. reported a similar finding, demonstrating that individuals with higher alexithymia traits (i.e., reduced emotional awareness, which is thought to be linked to lower interoception) exhibited a greater ability to inhibit imitation^22^. These studies suggested that interoceptive sensitivity or ability is positively correlated with greater overlap between self and other. In contrast, several studies using the imitation inhibition task^18^ and other measures of body ownership (rubber hand illusion^23^; enfacement effect^24^) reported that interoceptive sensitivity was positively correlated with a greater distinction between self and other (for a review, see^25^). Thus, several studies have suggested an association between interoception and the self-other boundary, but findings have been inconsistent, and the topic requires further examination.

Previous studies examining this issue have often used tasks involving imitation inhibition or the rubber hand illusion^21,23^. Therefore, it is possible that previous findings reflect task-dependent factors^25,26^. To avoid this issue, the present study focused on the phenomenon of spontaneous mimicry (task-independent contagion of body movement) to examine more implicit aspects of the self-other boundary and its relationship with interoception. Specifically, we examined spontaneous facial mimicry (SFM), in which observing another person’s facial expression leads to involuntary facial movements that match the perceived facial configuration^27–29^. We measured the frequency of SFM as an index of the degree of self-other boundary, and examined its correlation with interoception.

### Sensitivity to social cues

Another important function in social cognition is specific processing of socially relevant signals (hereafter referred to as “social cues”). Social cues are stimuli which indicate relevant information in interpersonal context, including other’s actions, faces, eye gaze, prosody, or touch. Humans are prone to prefer or spontaneously attend to these social cues^30–32^. As social cues indicate important information in interpersonal situations, specific processing of these signals is considered to be vital in development and learning of social cognition^33,34^. Importantly, Quattrocki and Friston argued that social cues trigger sensitivity to interoceptive signals, proposing that perceiving socially relevant stimuli causes the brain to put increased weight on interoceptive processing^8^. This weighting of interoceptive processing consequently promotes socio-affective inference and learning about the associated stimuli and/or situation. In this way, the researchers claimed that interoception plays a crucial role in developing and tuning social cognition. This influential claim, however, lacks direct evidence and remains to be tested. Given this background, the present study examined the possible association between interoception and sensitivity to social cues.

Eye gaze is a typical example of a socially relevant signal^35,36^, and whether or not the eyes of another person are directed to an observer carries important information. It has been well established that direct gaze (i.e., eye contact) attracts an observer’s attention and enhances positive social judgments of the other person^37,38^. This “eye contact effect” is also evident in SFM, in the way that mimicry is enhanced toward a model with direct gaze compared with averted gaze in relation to the observer^39,40^. In the context of the current study, we assumed that the degree of the effect of eye contact on SFM for an individual would indicate the extent to which they are influenced by social cues. In other words, we assumed that social cue sensitivity can be measured via the eye contact effect. Thus, the current study measured the eye contact effect and examined its correlation with interoception.

### Purpose and hypothesis of the current study

In summary, the current study aimed to elucidate the relationship between interoception and two factors underlying social cognition: self-other boundary and sensitivity to social cues. Specifically, we examined whether and how the performance of a heartbeat perception task (as an index of interoception) was associated with the frequency of SFM (as an index of self-other overlap) and the degree of eye contact effect on SFM (as an index of social-cue sensitivity). In addition, we conducted an alexithymia questionnaire and examined the correlations between the questionnaire score and the measures described above (see Appendix A for details).

In the present study, we measured participants’ interoception in terms of performance in the heartbeat counting task (i.e., silent counting of one’s own consciously perceived heartbeat^41^). This task is the most commonly used behavioural paradigm in interoception studies, and has been used in previous studies examining the relationship between interoception and the self-other boundary (e.g.,^18,21^). In the current study, we refer to this measure (i.e., performance on the heartbeat counting task) as “interoceptive accuracy” (IAc), following recent attempts to distinguish between behavioural- and questionnaire-based measures using different terminology for interoceptive “accuracy” and “sensibility”, respectively^42^.

Regarding mimicry and the effect of eye contact, the current study observed the SFM for movies in which models’ facial expressions change from neutral to smiling. There were two stimulus conditions, in which the gaze of models was directed or averted from the observers’ point of view. Using these settings, the self-other boundary was measured as the frequency of SFM in each condition. The degree of the eye contact effect was calculated as the difference in SFM frequency between the two conditions (direct vs. averted).

We assume that (1) If IAc is related to self-other overlap (or distinction), IAc would be positively (or negatively) correlated with SFM, and (2) If IAc is related to social-cue sensitivity, IAc would be positively correlated with the degree of the eye contact effect.

## Method

### Participants

The participants were 80 Japanese (45 males, mean age = 24.50 years, SD = 9.53, Range = 15.30–57.70). No participant reported having a history of psychiatric or neurological conditions.

### General procedure

All participants gave written informed consent that was approved by the Ethics Committee of The University of Tokyo (No. 18-50). All experiments and methods were performed in accordance with relevant guidelines and regulations. They first conducted a heartbeat counting task and then an SFM task. After these behavioural tasks, they responded to a self-report questionnaire.

### Heartbeat counting task

Participants were seated upright. They were instructed that “Please concentrate on your own heartbeat and count silently their consciously perceived heartbeats. The beginning and end of the counting periods are by start and stop tones. Do not take your pulse using hands and other parts of the body while doing this. Please report only felt heartbeats and do not estimate the number of heartbeats.” The task included four trials, with durations of 25, 35, 45, and 100 s, provided in a pseudorandomised order. They received no information on the duration of the trials or their performance. A wearable electrocardiogram-sensing system (myBeat; Union Tool, Co., Tokyo, Japan) monitored the participants’ heartbeats. The mean total time of the task was around 7 min. Data from two participants were excluded from the final analysis of this task due to experimenter error (n = 1) and inadequate execution, namely, counting the pulses visible in the hands (n = 1). The mean of heart beats was 74.40 bpm (*SD* = 10.44, *Range* = 51.32–100.60) in all participants.

The participants’ IAc scored were computed using the standard formula^43^: IAc = 1/4Σ[1 − (|recorded heartbeats − counted heartbeats|/recorded heartbeats)] * 100. The score range was 0–100, with 100 indicating veridical accuracy of heartbeat perception.

### SFM task

**Figure 1:**
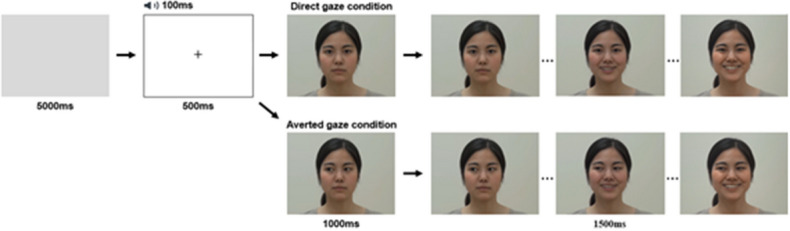
Examples of one trial in the direct- and averted-gaze conditions.

Participants were instructed to relax and observe the facial expressions on the display. They were shown video clips with an adult Japanese woman or man displaying a happy expression (Fig. 1). Each face stimulus was 9 cm in width and 13 cm in height on the display (visual angle = 11.421° × 16.438° each). All stimuli were edited using Adobe Premiere Pro CS6 (Adobe System Inc.). Four consecutive trials (one block) were presented in a fixed order (i.e., woman A, man A, woman B, man B) for each condition, for a total of 16 trials across 4 blocks. There were two conditions in terms of whether the gazes of the models in visual stimuli were directed to the participants (direct-gaze condition) or not (averted-gaze condition as a control condition). Half of the four blocks were exhibited the direct-gaze stimuli, and the remainder displayed averted gazes. The order of presentation for the four blocks was counterbalanced across participants. One trial lasted 2500 ms, and it was divided into two phases. In the first, in the direct-gaze condition, the model initially faced front and with a closed mouth and a neutral expression (1000 ms), and then a happy expression was displayed (1500 ms). In the averted-gaze condition, the stimuli were identical to those in the direct-gaze condition, with the exception that the model had her/his eyes averted. Neutral and happy facial expressions were included in a trial. Before each trial, a grey background was presented for 5000 ms. Then, a click sound was presented for 100 ms at the same time that a fixation point (indicated by a plus sign) was presented in the centre of a white background, for 500 ms. The mean total time of the task was around 2 min. During the videos, the participants’ facial reactions were recorded using a video camera.

Then, the facial reactions were coded by two trained coders who had no knowledge of the experimental conditions, using the Facial Action Coding System (FACS^44^). The action unit (AU; lip-corner pull, a prototypical facial action in a happy expression) was evaluated^29^. They also confirmed that participants were attending to the stimuli. The reliability of the first coder was tested by a second coder blind to the experimental conditions, who examined 25.0% of the data. Cohen’s kappa was high (0.925), confirming the reliability of the first coder. The analyses therefore used the data coded by the first observer only. SFM was considered to have occurred when a lip-corner pull was observed in the participant while the model was displaying a happy expression after a neutral expression. We set the participant’s facial expression as baseline while the model was displaying a neutral face. We calculated the total number of occurrence of SFM in each condition, and the difference score between the number of lip-corner pulls in the direct minus those in the averted condition were taken as an index of the eye contact effect (for an example of the same calculation of the eye contact effect, see^45^).

### Data analysis

We conducted a paired *t*-test, to investigate differences between the direct-gaze and the averted-gaze condition in the frequency of SFM.

The relations among all the variables (scores in the heartbeat counting task and SFM measures) were tested using Spearman rank correlation analyses, as this does not require any assumptions about the distribution of variables. The correlation analysis was conducted on 12 pairs of variables, therefore we applied a Bonferroni correction on them for the α-levels; α for the correlation analyses was 0.0042 = 0.05/12. (In fact, other methods for the correlation analysis, i.e. Pearson rather than Spearman, and other methods for controlling for multiple comparisons, i.e. Holm or FDR rather than Bonferroni, led to the same conclusions).

Furthermore, to examine the effect of gender, we conducted between-subject Welch’s *t*-test in performance on the heartbeat counting task, and a repeated-measures two-way ANOVA with “condition” (direct-gaze vs. averted-gaze) as a within-participants factor and “gender” (male vs. female) as between-participant factors in the frequency of SFM.

Regarding the SFM score, 34% of participants (27 out of 80) did not show facial mimicry in either the direct or the averted conditions. We report the whole data including these non-responders, confirming that all results from the correlation analyses do not alter even if these samples are included or excluded in the analyses.

## Results

### Heartbeat counting task

The mean value for task performance was 67.95 (*SD* = 19.43, *range* = 27.41–98.25) in the total sample (*N* = 78). It showed no significant correlation with participants’ heart rate (*ρ* (76) = − 0.184, *p* = 0.106, 95% CI [− 0.39, 0.041]).

### SFM task

Within-subject *t*-test confirmed that the frequency of SFM in the direct-gaze condition was significantly higher than the averaged condition (*t* (79) = 4.501 *p* =  < 0.001, *r* = 0.45).

### Relationships between IAc and SFM

**Figure 2:**
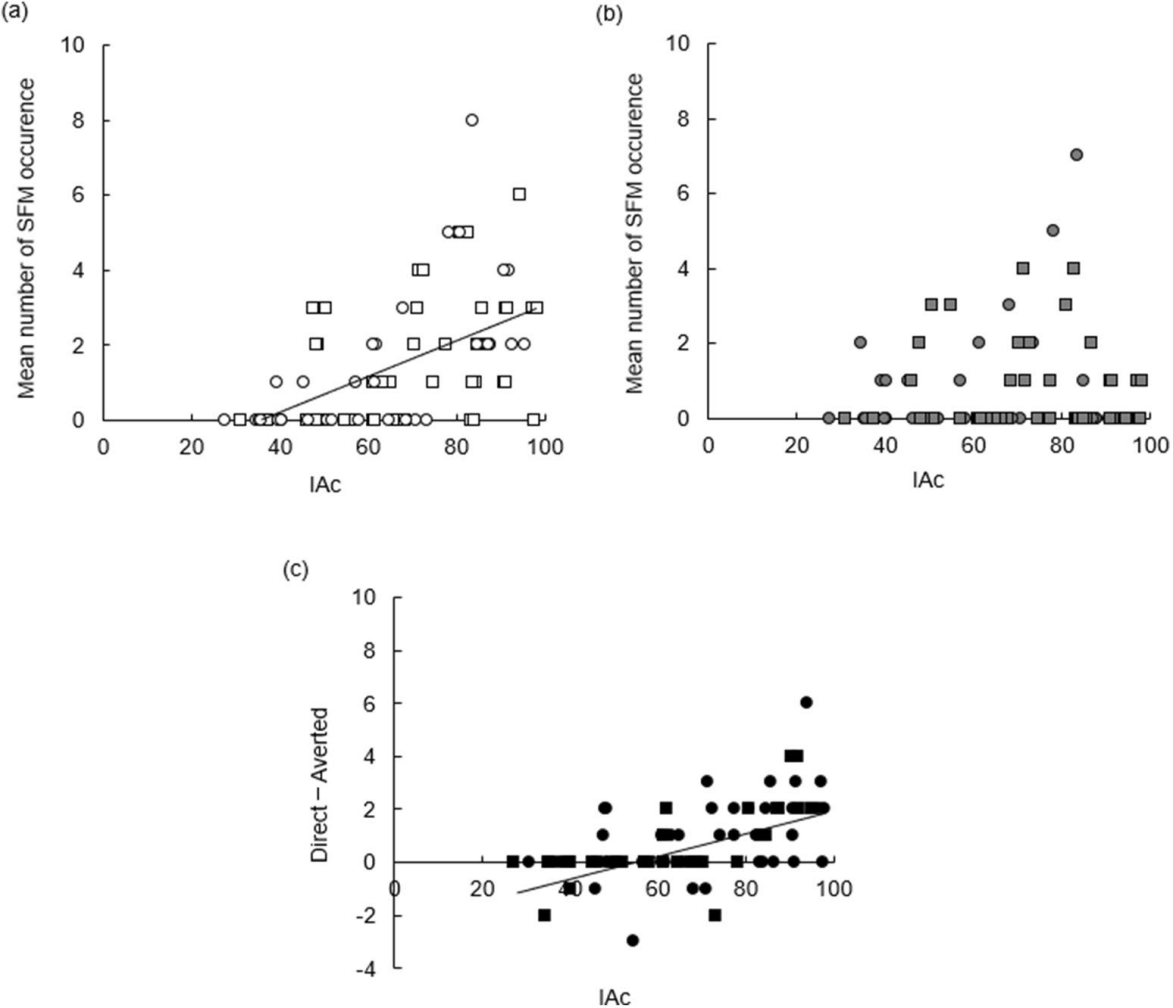
Correlation between IAc and the mean occurrences of SFM in the (**a**) direct-gaze and (**b**) averted-gaze conditions and (**c**) between IAc and the difference in scores between the direct- and averted-gaze conditions. Square dots represent male data, and circular ones represent female data.

In the direct-gaze condition, a significant positive correlation was found between the performance of the heartbeat counting task (IAc) and the mean number of occurrences on SFM (*ρ* (76) = 0.574, *p* < 0.001, 95% CI [0.40, 0.71]; Fig. 2a), while the correlation was not significant in the averted condition (*ρ* (76) = 0.030, *p* = 0.794, 95% CI [− 0.19, 0.25]; Fig. 2b). Importantly, we found a significant positive correlation between IAc and the difference in occurrences of SFM between the direct-and averted-gaze condition (*ρ* (76) = 0.603, *p* < 0.001, 95% CI [0.43, 0.73]; Fig. 2c).

### Examination in each gender

We checked if any results reported above were differentiated, or influenced, by the factor of gender of participants. Between-subject Welch’s *t*-test showed that IAc (the scores of heartbeat counting task; males, *M* = 71.25, *SD* = 18.00, *range* = 31.00–98.25; females, *M* = 63.44, *SD* = 20.65, *range* = 27.41–95.43) were not significantly different between genders (*t* (76) = 1.780, *p* = 0.079).

**Figure 3:**
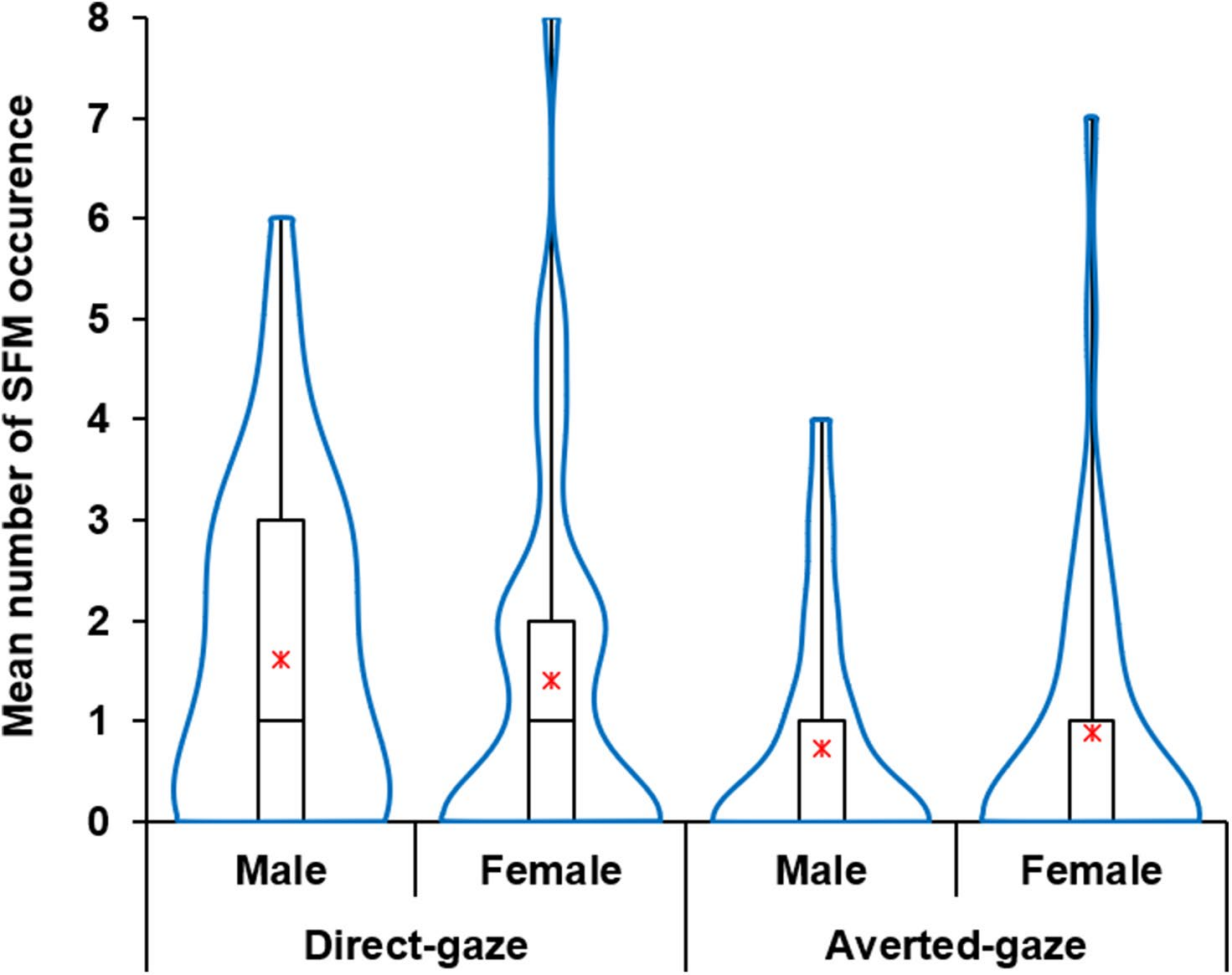
Box and Violin plots for the number of occurrences of spontaneous mimicry in the SFM task (*N* = 80) for each gender.

Two-way ANOVA (condition × gender) showed a significant main effect of condition on occurrences of SFM (Fig. 3; see also Appendix B for plots of individual data), in which more SFM was observed in the direct-gaze condition than in the averted-gaze condition (*F* (1, 78) = 18.758, *p* < 0.001, *η*_*p*_^2^ = 0.19). The analysis did not yield a significant effect for gender (*F* (1, 78) = 0.012, *p* = 0.912, *η*_*p*_^2^ = 0.00) or interaction between condition and gender (*F* (1, 78) = 1.337, *p* = 0.251, *η*_*p*_^2^ = 0.02).

The correlations among measures were tested in each gender group separately. The results were identical to the overall data: in both gender groups, significant correlations were detected in the differences in scores (direct vs. averted conditions) (males, *ρ* (43) = 0.461, *p* = 0.001, 95% CI [0.19, 0.66]; females, *ρ* (31) = 0.688, *p* < 0.001, 95% CI [0.45, 0.83]), as well as in the direct-gaze condition (males, *ρ* (43) = 0.430, *p* = 0.003, 95% CI [0.16, 0.64]; females, *ρ* (31) = 0.562, *p* = 0.001, 95% CI [0.27, 0.76]), but not in the averted-gaze condition (males, *ρ* (43) = 0.021, *p* = 0.889, 95% CI [− 0.27, 0.31]; females, *ρ* (31) = 0.160, *p* = 0.366, 95% CI [− 0.19, 0.48]). As such, all the results of correlation analyses on the overall data were equally observed in each gender group.

## Discussion

### Summary of the findings

The present study examined whether and how the interoceptive accuracy (IAc, indexed by the performance of heartbeat counting task) is associated with the degree of self-other overlap/distinction (indexed by the SFM frequency) and the sensitivity to socially relevant stimuli (indexed by the degree of eye contact effect on SFM). The main results can be summarised as follows: (1) the correlation between SFM frequency and IAc varied between conditions, with a positive correlation only in the direct gaze condition; (2) the extent of eye contact effect was positively correlated with IAc; (3) these results in the overall sample were also exhibited in separate analyses of male and female participant groups, supporting the robustness of the main findings.

### Self-other boundary

If the frequency of SFM had been correlated with IAc irrespective of the condition, the interoceptive sensitivity could be considered to be positively associated with the tendency of self-other overlap. However, the results revealed that the correlation depended on the eye-gaze condition, with no significant correlation in the averted gaze condition. There are several possible interpretations of this result. One possibility is that the direct gaze condition corresponded to participants’ “default” condition, and the relationship in the averted condition was distorted for some reason. This interpretation would imply that the two factors (i.e., self-other overlap and IAc) are positively associated, although something exceptional happened in the averted condition. Another possibility is that the averted condition represented the normal situation, and the significant correlation in the direct condition was just the overlaying of the eye contact effect, which actually exhibited a significant positive correlation. This interpretation implies that the two factors are not related. Unfortunately, the design of the present study did not allow us to test which of these explanations is more valid. As mentioned in the introduction of this paper, previous studies reported contradictory results; some studies reported that IAc was positively correlated with the lower interpersonal boundary (greater overlapping)^21,22^ and some reported it with the higher boundary (greater distinction)^18,25^. The present findings indicated that the correlation of IAc with the self-other boundary, if any, would be with the lower boundary (i.e., the amount of overlapping, not distinction) between self and other. This may be the case, at least when the interpersonal boundary is assessed in an involuntary manner as in the current study, rather than under goal-directed tasks as in previous studies. The possible influence of the nature of different tasks should be examined in future.

### Social cue sensitivity

Compared with the self-other boundary, examination of the eye contact effect provided relatively clear results. First, the mean frequency of SFM was significantly higher in the direct gaze condition compared with the averted condition at the group level, confirming that eye contact did enhance mimicry^39^. Importantly, the current results revealed individual differences: the magnitude of the eye contact effect was positively correlated with performance on the heartbeat counting task (IAc). That is, higher IAc corresponded to greater impact of eye contact. This result suggests that IAc is related to sensitivity to social cues (or socially relevant conditions).

“Sensitivity” can be functionally explained in terms of arousal or attention. Direct gaze is known to activate the observer’s arousal more strongly compared with averted gaze^36,46,47^. Generally, increasing arousal enhances response to stimuli. Thus, one explanation of the current result is that individuals with higher IAc may also exhibit greater arousal in response to the direct gaze, consequently exhibiting a greater eye contact effect. In conjunction with the arousal response, which is likely to illustrate a passive aspect of the process, a more active account can be also considered in terms of top-down attentional processes. That is, the greater impact of eye contact may indicate a greater amount of resource allocation or selective weighting in the processing of others’ direct gaze.

In a related study, Isomura and Watanabe reported that viewing direct gaze enhanced IAc, which was measured by the heartbeat counting task (although this effect was observed only in participants with low IAc)^48^. Heart rate did not differ between direct and averted gaze conditions, suggesting that their results was not likely to be due to arousal-related factors. Although this previous study and the present study are not directly connected, the findings of both studies indicate the existence of an association between social cue (gaze) perception and interoception.

### Alternative interpretations of the results

So far, we have assumed that the SFM data (those of the direct and averted conditions and its difference) reflect specific functions (i.e. self-other boundary and social cue sensitivity, respectively). However, other functions can be indicated by the SFM measures. For example, it is possible that SFMs in each condition were not the consequence of self-other matching, but they reflected the positive emotion which is evoked by the emotional stimuli (in this case, another’s smile). This possibility implies that the result can indicate the degree of general emotional response to another’s facial expression. Actually, previous studies have suggested that individuals with higher IAc tend to show stronger emotional responses to affective visual stimuli^43,49^.

The difference between the direct and the averted conditions may also reflect functions other than that of our assumption. Niedenthal et al. argued that the facial mimicry is amplified in the condition of eye contact because it triggers deep processing, specifically, embodied (corporeal) simulation, of perceived facial expression^50^. Following this view, the difference score of SFM can be considered as a strength of the trigger for deep internal processing, or the magnitude of embodied simulation. As such, we note that there can be various accounts for functions which are represented by the current data/results.

### Future directions

Finally, we discuss several approaches that may be useful for further elucidating the contribution of interoception to the two factors examined in the current study.

Regarding the relationship between interoception and the self-other boundary, previous studies have reported mixed findings as mentioned above, and the present study failed to provide sufficiently clear evidence. The current findings suggested that interoception is associated with contextual modulation of the self-other boundary (reflected by the correlation between IAc and eye contact effect on SFM). This further suggests that it is important to examine not only the direct association between interoception and the self-other boundary, but also the possible indirect contribution of interoception (as a “hidden factor”) under contextual modulation of the self-other boundary. It has been reported that spontaneous mimicry is influenced not only by eye gaze, but also by various types of contextual information, including familiarity or similarity between observer and target, and task demands^51,52^. Similar influences have also been reported in explicit tasks, including automatic imitation^21^ and judgment of body ownership^23^. Therefore, future studies should clarify whether such contextual effects on the self-other boundary are modulated by interoceptive factors.

Regarding social cue sensitivity, the present findings require further validation using various types of social cues and/or tasks. Considering that the present findings suggested a possible interaction between self-other boundary and social cue sensitivity, a potentially useful approach may be to examine social cue sensitivity independently of the self-other boundary. For example, examining the relationship between interoception and social cue sensitivity by measuring the amount of attentional bias^53,54^, the threshold of conscious detection^38^, or the impact on memory^55,56^ in direct gaze compared with averted gaze conditions could provide further confirmatory data.

In addition, further studies should also use neural-physiological measures. For example, measuring electrodermal activity may be useful for assessing the potential role of arousal-related factors discussed above. Neurophysiological measurement of interoception, such as heartbeat-evoked neural response, is also a promising approach. Several previous studies reported that heartbeat-evoked response is associated with social processing, such as introspection of self^57^ or judgment of others’ facial expression^10^. The combination of these measures may allow us to examine individuals’ interoceptive and social processing separately from general arousal- and attention-related factors.

## Supplementary information

Supplementary Information.

## Data Availability

All data analysed during this study are available from the corresponding author on reasonable request.
