# Supplementary material for: Interoception is associated with the impact of eye contact on spontaneous facial mimicry
Source: Sci Rep. 2020 Nov 16;10:19866. doi: 10.1038/s41598-020-76393-8 (PMC7670470; doi:10.1038/s41598-020-76393-8)
Supplement: Supplementary file 1 — Supplementary Information. [file 41598_2020_76393_MOESM1_ESM.docx]

**Interoception is associated with the impact of eye contact on spontaneous facial mimicry**

Masahiro Imafuku*, Hirokata Fukushima*, Yuko Nakamura, Masako Myowa, Shinsuke Koike

**Appendix A: Examination of a possible association with alexithymia**

As an additional research question, we examined a possible association between alexithymia and the factors examined in this study (i.e., interoceptive accuracy [IAc], spontaneous facial mimicry [SFM], and the degree of eye contact effect on SFM). Alexithymia is a condition/trait involving difficulty or reduced ability in emotional recognition (Sifneos, 1973). In accord with several theories assuming that emotional awareness is rooted in bodily (especially interoceptive) awareness (James, 1884; Seth, Suzuki, & Critchley, 2011; Barrett & Simmons, 2015), alexithymia and interoception are thought to have important associations, with some previous studies suggesting that alexithymia is correlated with lower interoception (e.g., Murphy, Catmur, & Bird, 2018). As described in the Introduction, Sowden, Brewer, Catmur, and Bird (2016) reported that higher alexithymia traits were associated with greater ability to inhibit imitation (i.e., higher self-other distinction). Based on this background, we measured participants’ alexithymia traits using a questionnaire and investigated correlations with the measures in the main study.

**Method**

Participants responded to the Japanese version of the Toronto Alexithymia Scale (Japanese TAS-20, Komaki et al. 2003). The TAS-20 includes 20 items, each rated on a 5-point Likert scale, with total scores ranging from 20–100. Higher scores in the TAS-20 indicate higher trait alexithymia. One participant did not respond to the questionnaire, and their data were excluded from the correlation analyses.

**Results**

Alexithymia questionnaire. The total TAS-20 score (*M* = 52.70, *SD* = 9.40, range = 30–72) was not correlated with the IAc (ρ [75] = −0.104, *p* = .370), the mean occurrence of SFM in the direct- or averted-gaze conditions (ρ [77] = −0.001, p = .995; ρ [77] = −0.034, *p* = .766) or the difference scores (ρ [77] = 0.030, *p* = .791). Overall, TAS-20 scores did not show any correlations with other variables in the current study.

**Discussion**

Our data did not provide any evidence for an association between alexithymic traits and IAc, self-other boundary, or social-cue sensitivity. Although the factorial structure of the questionnaire (Japanese TAS20) did not exhibit a unique pattern compared with the original TAS20 (Bagby, Parker, & Taylor, 1994), there could have been culture-specific influences in the correlation with some behavioural data. Further investigation is required to clarify how the tendency of affective experience in daily life interacts with the association between interoception and social cognition.

**References**

Bagby, R. M., Parker, J. D., & Taylor, G. J. (1994). The twenty-item Toronto Alexithymia Scale--I. Item selection and cross-validation of the factor structure. *J. Psychosom. Res*, *38*(1), 23–32.

Barrett, L. F., & Simmons, W. K. (2015). Interoceptive predictions in the brain. *Nat. Rev. Neurosci*, *16*, 419–429.

James, W. (1884). What is an emotion? *Mind*, *9*, 188–205.

Komaki, G., Maeda, M., Arimura, T., Nakata, A., Shinoda, H., & Ogata, I., et al. (2003). The reliability and factorial validity of the Japanese version of the 20-Item Toronto Alexithymia Scale (TAS-20). *Jpn. J. Psychosom. Med*, *43*, 839–846.

Murphy, J., Catmur, C., & Bird, G. (2018). Alexithymia is associated with a multidomain, multidimensional failure of interoception: evidence from novel tests. *J. Exp. Psychol. Gen*, *147*(3), 398–408.

Seth, A. K., Suzuki, K. & Critchley, H. D. (2011). An interoceptive predictive coding model of conscious presence. *Front. Psychol*, *2*: 395.

Sifneos, P. E. (1973). The prevalence of “alexithymic” characteristics in psychosomatic patients. *Psychother Psychosom*, *22* , 255–262.

Sowden, S., Brewer, R., Catmur, C., & Bird, G. (2016). The specificity of the link between alexithymia, interoception, and imitation. *J. Exp. Psychol. Hum. Percept. Perform*, *42*, 1687–1692.

**Appendix B: Individual data points for the data depicted in Figure 2**


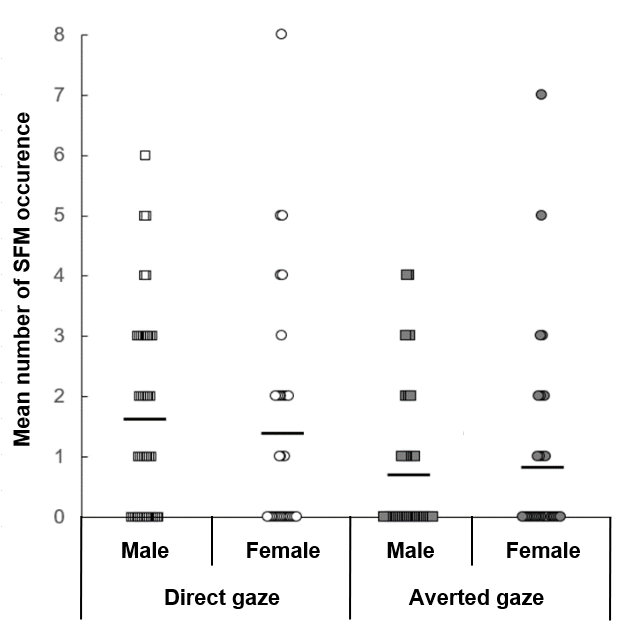


Figure A1: Individual data plots for the number of occurrences of spontaneous mimicry in the SFM task for each gender. The data were identical to those in Figure 2 in the main text. Horizontal bars represent the mean values.
